# Editorial: Advances in fruit-growing systems as a key factor of successful production, volume II

**DOI:** 10.3389/fpls.2026.1831745

**Published:** 2026-03-25

**Authors:** Geza Bujdoso

**Affiliations:** Institute for Horticultural Sciences, Fruit Growing Research Centre, Hungarian University of Agriculture and Life Sciences, Godollo, Hungary

**Keywords:** determination of ripening, plant health, plant nutrition for dragon fruit, plant protection, rootstock/scion interactions for mango

The fruit production industry currently faces numerous challenges, including climate change, the introduction of new fruit-bearing species, the traits of newly bred cultivars, innovations in orchard systems, rootstock–scion interactions, the impact of modern cultivation technologies on yield, the emergence of new pathogens and pests, bird and mammal interference in orchards, the development of successful organic production systems, fruit quality and composition, regulatory frameworks, high labor demands, and the cultivation of new fruit cultivars. Farmers require timely and accurate information to address these complex and continuously evolving issues. Within the framework of the Research Topic *“Advances in Fruit-Growing Systems as a Key Factor of Successful Production: Volume II,”* seven scientific papers were accepted.

Determining the optimal harvest time is not always easy for fruit species that lack clear visual indicators on the fruit surface. The accepted scientific paper titled *“Lightweight MSW-YOLOv8n-Seg: Instance Segmentation of Maturity in Cherry Tomato with Improved YOLOv8n-Seg”* presents a promising model that can assist in determining the optimal harvest time for cherry tomato. Another accepted original research article, *“Maturity Detection and Counting of Blueberries in Real Orchards Using a Novel STF-YOLO Model Integrated with the ByteTrack Algorithm,”* also focuses on harvest time determination. The developed algorithm is suitable for identifying the optimal harvest time under real orchard conditions. Furthermore, the accepted original research article *“Genome-wide Analysis of the AP2/ERF Family in Akebia trifoliata and Characterization of the AtrERF001 Gene Regulating Fruit Ripening”* investigates the genetic regulation of fruit ripening on *Akebia trifoliata*. Ripening in this species is closely associated with ethylene response factor genes. The researchers identified 131 genes in total and concluded that nine key ethylene response factor genes play an important role in the ripening process of this species.

Plant health represents another major topic in this Research Topic. The accepted original research article *“Delaying Candidatus Liberibacter asiaticus Infection of Citrus Trees Through the Use of Individual Protective Covers and Systemic Delivery of Oxytetracycline”* examines strategies to protect citrus trees against this phloem-limited bacterium. The combined use of oxytetracycline (OTC) delivered via trunk injection and Individual Protective Covers (IPCs) proved to be highly effective. This combined treatment significantly influenced tree size, fruit yield, fruit components, peel color, juice yield, and canopy health, regardless of previous infection history or rootstock.

The accepted brief report titled *“Mortality of Apricot Rootstocks and Scions”* investigates the effects of rootstocks and scions on several traits, including tree mortality during the non-bearing years. Two vigorous rootstocks (‘Montclar’ and ‘Rootpac R’) performed well under Hungarian climatic and soil conditions in lowland areas and showed lower mortality compared with the moderately vigorous rootstocks examined (‘Fehér besztercei’ and ‘Wavit’). The presence of a strong root system plays a dominant role in this regard.

Plant health is also closely linked to plant nutrition; therefore, determining optimal nutrient supply for different fruit species and production sites is essential. The accepted original research article *“Optimization of Fertilizer Doses for Increased Fruit Yield of Dragon Fruit Using Response Surface Methodology (RSM) and Box–Behnken Design”* provides an excellent example of optimizing nutrient supply for dragon fruit cultivation. According to the results of the Indian researchers, the optimum fertilizer doses (N:P_2_O_5_:K_2_O) were 400:300:650 g/pillar/year and 700:400:350 g/pillar/year for white-fleshed and purple-red-fleshed dragon fruit, respectively. These application rates resulted in maximum yields of 25.5 t ha^−1^ and 35.6 t ha^−1^ for white- and purple-red-fleshed dragon fruit, respectively.

Rootstock–scion interactions remain an important Research Topic in fruit production. This Research Topic is particularly relevant for fruit species with limited production potential due to climatic or soil constraints. The use of ‘Olour’ as both rootstock and scion, as well as ‘Mallika’ and ‘Amrapali’ as interstocks for mango (*Mangifera indica* L.), was examined in one of the papers included in this Research Topic. According to the results of the Indian research group, the Olour/Mallika/Olour combination performed best among the studied interstock combinations. Among the single-graft combinations, Amrapali/Olour exhibited moderate vigor, whereas the Mallika/Olour combination showed high vigor and favorable growth traits.

This Research Topic provides a diverse collection of newly developed results related to fruit-growing systems applied worldwide. As natural conditions vary greatly among production regions, the most effective solutions and optimal practices may differ from one fruit-growing site to another.

